# The Role of Canalization and Plasticity in the Evolution of Musical Creativity

**DOI:** 10.3389/fnins.2021.607887

**Published:** 2021-03-16

**Authors:** Piotr Podlipniak

**Affiliations:** Department of Musicology, Adam Mickiewicz University in Poznań, Poznań, Poland

**Keywords:** canalization, plasticity, gene-culture coevolution, musical creativity, musical syntax, cortico-subcortical loops, premotor cortex

## Abstract

Creativity is defined as the ability to generate something new and valuable. From a biological point of view this can be seen as an adaptation in response to environmental challenges. Although music is such a diverse phenomenon, all people possess a set of abilities that are claimed to be the products of biological evolution, which allow us to produce and listen to music according to both universal and culture-specific rules. On the one hand, musical creativity is restricted by the tacit rules that reflect the developmental interplay between genetic, epigenetic and cultural information. On the other hand, musical innovations seem to be desirable elements present in every musical culture which suggests some biological importance. If our musical activity is driven by biological needs, then it is important for us to understand the function of musical creativity in satisfying those needs, and also how human beings have become so creative in the domain of music. The aim of this paper is to propose that musical creativity has become an indispensable part of the gene-culture coevolution of our musicality. It is suggested that the two main forces of canalization and plasticity have been crucial in this process. Canalization is an evolutionary process in which phenotypes take relatively constant forms regardless of environmental and genetic perturbations. Plasticity is defined as the ability of a phenotype to generate an adaptive response to environmental challenges. It is proposed that human musicality is composed of evolutionary innovations generated by the gradual canalization of developmental pathways leading to musical behavior. Within this process, the unstable cultural environment serves as the selective pressure for musical creativity. It is hypothesized that the connections between cortical and subcortical areas, which constitute cortico-subcortical circuits involved in music processing, are the products of canalization, whereas plasticity is achieved by the means of neurological variability. This variability is present both at the level of an individual structure’s enlargement in response to practicing (e.g., the planum temporale) and within the involvement of neurological structures that are not music-specific (e.g., the default mode network) in music processing.

## Introduction

Music has been observed in all human cultures and has accompanied our ancestors since the dawn of our species (Morley, 2013), or perhaps even earlier as part of ancient hominin behavior (Mithen, 2006). Therefore, musicality as a set of abilities that enable music production and recognition (Fitch, 2015), is often claimed to be a product of biological evolution (Roederer, 1984; Hagen and Bryant, 2003; Peretz, 2006; Hagen and Hammerstein, 2009; Mithen, 2009). As music has its roots in the vocal behavior of our ancestors (Bannan, 2012; Morley, 2013), the evolution of musical creativity cannot be explained without tracing the beginnings of hominin vocal communication. Singing, the vocal form of musical activity, belongs together with crying, laughter and speech, as a group of characteristic vocalizations of *Homo sapiens*, and is often compared with songbirds’ songs (Merker, 2005; Fitch and Jarvis, 2013; Rothenberg et al., 2014) or the songs of other species (Geissmann, 2000; Payne, 2000). Human and songbird songs are good examples of ritual culture because the structures of their songs are the objects of imitation by conspecifics (Merker, 2009). In order to achieve this trait, the evolution of vocal learning among songbirds and hominins was necessary. Another important similarity between human and songbird songs is that both humans and songbirds modify their songs so that new versions become an object of cultural transmission. This means that apart from an urge to imitate a song, individuals are also prone to invent at least some new structural elements. This task necessitates both the knowledge of rules that govern a particular song style and creativity, as not all possible sound variants can be recognized as a song and therefore appreciated by conspecifics. From this point of view, musical creativity, similar to songbird vocal creativity, should not only have the ability to generate something novel and original in the domain of music, but also have something valuable (Merker, 2006). The invention of any new musical piece must be a result of the “creative thinking process” which in music can be viewed as consisting of three stages: (i) product intentions, (ii) the thinking process, and (iii) the creative product (Webster, 1990, 2002). While the product intentions phase is strictly related to the kind of urge that drives a creator to musical activity (e.g., composition, performance, improvisation) the thinking process is based on divergent and convergent thoughts (Webster, 2002). Divergent thoughts involve the imagination of musical elements such as melodic or rhythmic motives (Webster, 2002) which can occur involuntarily (Copeland, 2019). In contrast, convergent thoughts seem to be analytical and lead to aesthetic decisions (Webster, 2002). Only after this is the final creative product achievable. Since the result of an effective creative process in music is usually positively assessed by a social environment, being creative gives an advantage to creative individuals. Therefore, musical creativity can be viewed as a biological adaptation. As every creative act operates on the existing cultural information, musical creativity can also be understood as “an adaptive process of knowledge acquisition” (Reybrouck, 2006). However, while creativity in general refers to the ability that allows us to use all possible cognitive resources, the creative process in the domain of song is restricted by the scope of sound features which characterize conspecific songs. Therefore, in contrast to general creativity that is often understood as being free from any limitations, the evolutionary beginnings of creative abilities in the domain of song seem to be tightly connected to a domain-specific form of communication.

The fact that humans are so constrained by music-specific features (Harwood, 1976; Nettl, 2000; Brown and Jordania, 2011; Savage et al., 2015; Mehr et al., 2019) and at the same time so creative while inventing the variations of a sound sequence, suggests that human musicality must be based on at least two antithetical predispositions. One impels humans to be as precise as possible in copying musical distinctive sound features. This copying capacity is possible thanks to a human ability unique among primates to vocally learn (Janik and Slater, 1997; Merker, 2005, 2012), especially the sounds of speech and singing (Bannan, 2008). The other predisposition allows us to be inventive enough to create sound sequences that are ear-catching for the other members of a social group. Without this ability, no variations of music, and thus any cultural evolution of music, would be possible (Savage, 2019). By taking into account the contrastive character of the aforementioned abilities, a multistage evolutionary scenario must be considered to explain the origin of musical creativity. After all, it is hard to imagine how such a complex variability of human songs could have evolved as a result of one accidental event. Instead, the appearance of musical creativity as we know it today must have been evolving gradually in response to many evolutionary pressures. On the one hand, these pressures must have led to the appearance of music-specific perceptive, functional and behavioral biases (Mehr et al., 2019). On the other hand, these biases must not have been strong enough to restrict the scope of hominins’ vocal repertoire to a closed set of calls. The result is *Homo sapien* musical culture which can be characterized by the influence of at least four levels of constrains: (1) inherited perceptive and behavioral biases which influence the existence of musical universals; (2) enculturated (culturally inherited) biases which consists of implicitly learned elements of a musical system such as culture-specific pitch intervals and rhythm ratios; (3) limitations of creativity which are related to the efficiency of the brain, restricted for instance, by the capacity of working memory; (4) social selective pressures which act as feedback able to modify former constraints in the long run. The existence of all these constraints indicates that gene-culture coevolution (Lumsden and Wilson, 1980) could have been an evolutionary mechanism leading to the origins of human musicality (Podlipniak, 2015, 2017; Killin, 2016, 2017; Patel, 2018; Savage et al., 2020). It is worth mentioning, that all these aforesaid constraints act simultaneously and must have been present at all stages of our gene-culture coevolution, which has led to the emergence of human musical culture. Therefore, the evolution of musical creativity, being an inseparable part of this process, must have been influenced, at least, by some of the same evolutionary forces that have shaped the evolution of human musicality.

This paper takes a theoretical and naturalistic approach in order to explain the evolutionary roots of musical creativity. Since musical creativity is based on innovative thinking in the domain of sound communication, the presented framework is focused only on the relatively recent evolutionary history of the *Homo* lineage in which vocal learning started to play an important role, rather than depicting the whole evolutionary origin of human musicality. We begin with the idea of gene-culture coevolution and its aforementioned two main evolutionary mechanisms—plasticity and canalization. The next section is devoted to the conditions that influenced the evolution of musical creativity, in which the point of departure for the coevolutionary pathways leading to emergence of human musicality is demonstrated, including details concerning the possible interplay between canalized and innovative factors in the domain of pitch and rhythm. In the following section, the view of musical syntax as a canalized framework for musical creativity is discussed. In the conclusion the importance of musical creativity for the evolution of human musicality is emphasized, and possible future research that can contribute to the evolutionary explanation of the origin of musical creativity is suggested.

## Plasticity and Canalization as the Main Mechanisms of Gene-Culture Coevolution

In general, gene-culture coevolution is a circular process in which genetic information influences culture whilst also simultaneously changing in response to culture that acts as a selective environment (Lumsden and Wilson, 1982). The influence of genetic information on culture can be achieved by different mechanisms related to either somatic characteristics or behavioral proclivities. Wilson (Wilson, 1978) described this influence metaphorically by saying that “the genes hold culture on a leash” (p. 167). These mechanisms restrict the type of cultural information to be exchanged (e.g., the dominance of visual and acoustic cues as the means of communication in primates) but they also allow a species to create a species-specific cultural niche (e.g., agriculture). However, apart from being under such constraints many animals are able to socially learn (Laland, 2017) which makes them flexible at overcoming new challenges that can appear in a fast-changing environment. Such flexibility is especially useful in a complex and often fast changing cultural environment that characterizes human culture. Nonetheless, not all cultural traits change equally fast. When a particular cultural environment is stable enough, i.e., it lasts throughout many generations; it can become a source of selection. As a consequence, such a stable cultural environment can lead to the appearance of traits that are under genetic control. The clearest example of gene-culture coevolution is the evolution of lactase persistence (Gerbault et al., 2011). It is assumed that the domestication of animals by humans created a pastoral cultural niche which became a selective environment for the appearance of lactose tolerance among adults about 8,000 years ago (Leonardi et al., 2012). As a result, a population dominated by individuals characterized by lactose tolerance has been prone to sustain and elaborate the practice of dairying. In this example, a cultural invention—farming—became a starting point for a chain of interdependent coevolutionary events. Similar coevolutionary mechanisms have been proposed for the evolution of many human traits such as handedness, mating preference (Laland, 2008), language (Dor and Jablonka, 2000, 2001; Bickerton, 2010; Deacon, 2010) and music (Podlipniak, 2017; Patel, 2018; Savage et al., 2020). Music in gene-culture coevolution was initially invented by hominins and by having an adaptive function, music abilities have been preferred by natural selection which has strengthened and accelerated the evolution of human musicality. According to Patel for example, the invention of music had unexpected adaptive effect—social bonding, which was the cause of the coevolutionary chain of circumstances (Patel, 2018). In another coevolutionary scenario of music evolution (Podlipniak, 2017), the social bonding function was an attribute of music right from the beginning. In all these scenarios, however, genes and culture has been permanently interacting.

### Plasticity

The necessary condition for gene-culture coevolution is the ability of phenotypes to modify behavior in response to the environment. This kind of response is often called behavioral plasticity (Dor and Jablonka, 2010; Mery and Burns, 2010), a form of developmental (or phenotypic) plasticity (Pigliucci, 2001; Fusco and Minelli, 2010) which, along with genetic variation, is the property of all living organisms (West-Eberhard, 2005). Thanks to plasticity every phenotype can be flexible to some extent, which allows it to adapt in response to environmental changes during its lifetime. For behavioral plasticity to occur a phenotype has to learn. Although behavioral plasticity and learning are often associated with neuronal plasticity, both can be achieved by other mechanisms too (Jablonka and Lamb, 2005; Mery and Burns, 2010). It must be emphasized, however, that developmental, including behavioral, plasticity being the terms that refer to the functional explanation of the adaptation to the environment (the survival value of a trait), belongs to Tinbergen’s ultimate level of explanation (Tinbergen, 1963) which focuses on the phylogeny and function of a phenotypic trait (Fitch, 2015). In contrast, neuronal plasticity as a physiological mechanism that allows the brain to modify the preexisting neuronal connections in response to changes in afferent inputs or efferent requirements (Pascual-Leone et al., 2005) represents Tinbergen’s proximal level of explanation (Tinbergen, 1963), which concerns the mechanistic and developmental elucidations of how a phenotypic trait works and develops in ontogeny (Fitch, 2015). Therefore, although behavioral plasticity can be achieved by the means of neuronal plasticity, only the former refers to the gene-culture coevolutionary explanations of the appearance of musical creativity.

From this perspective plasticity is a crucial process that enables cultural change. It seems that plasticity has played a crucial role in the evolution of humans, since our culture is cumulative (Tomasello, 1999), which means that cultural traits such as behaviors, technologies and ideas are not only learned and transmitted from generation to generation but also modified and improved so that new elements are often added to the existing ones. An increasing number of innovations and modifications lead to the appearance of a rich cultural environment which undergoes its own evolution. The process of cultural evolution resembles to some extent genetic evolution (Creanza et al., 2017), since in both cases the gradual process of transmission and modification of traits by means of natural selection is observed. This means that all cultural traits have to compete to reproduce (cultural transmission). However, in contrast to genetic evolution in which variations of traits are achieved by means of mutations and recombination, at the level of cultural evolution plasticity is a source of diversity. Moreover, without plasticity the interaction between cultural and genetic evolution would be impossible. After all, thanks to plasticity the first Anatolian farmers were able to invent a dairy based diet which started the gene-culture coevolution of the aforementioned lactase persistence.

### Canalization

The concept of “canalization” was originally proposed by Waddington (1942) and refers to “the ability of a genotype to produce relatively constant phenotypes regardless of environmental and genetic variabilities” (Takahashi, 2019, p. 14; cf. also Dor and Jablonka, 2010, p. 138; Loison, 2019, p. 6). Importantly, such constancy concerns not only somatic but also behavioral traits. Canalization can be achieved by different means including cultural and genetic control over a given trait. A good example of an evolutionary mechanism related to gene-culture coevolution in which canalization plays a crucial role is the Baldwin effect (Baldwin, 1896a,b; Sznajder et al., 2012). In the first step of the Baldwinian mode of evolution, organisms adapt to the environmental challenges by means of phenotypic adaptation. In the case of culturally achieved phenotypic adaptation, adaptive learning plays a crucial role. Thanks to genetic variability of a population, sooner or later a new genotype appears and endues an individual with an instinct allowing the replacement of phenotypic adaptation with genetic adaptation (Dor and Jablonka, 2000). If this learning is costly, i.e., it needs a lot of energy and/or is time-consuming, such an individual is favored by natural selection and in the long run the whole population becomes dominated by individuals endowed with this instinct. In other words, in these circumstances a trait that is initially achieved by plastic response to environmental pressure is followed by a genetic change which takes control over the previously culturally achieved trait. The ability to develop this trait, independent of whether it is achieved by learning or genetic control, represents an example of canalization. Different circumstances can promote either plasticity or canalization. While plasticity is an effective mechanism for adaptation to a fast-changing environment, canalization can be viewed as a safety-valve based on evolutionary “memory.” As such, canalization can be understood as “the buffering of environmental and genetic < < *noise*> >” (Dor and Jablonka, 2010, p. 135).

## The Evolution of the Musical Mind and Musical Creativity

The processing of music is a complex task which consists of many sequential and simultaneous computations that reflects different adaptive mechanisms of a different evolutionary age. Only a small part of these processes can be viewed as music-specific. The vast majority of these mechanisms fulfill more general functions related to audition, including auditory scene analysis such as the detection and recognition of sound sources (Bregman, 1990), and various forms of acoustic communication (Zimmermann et al., 2013; Ackermann et al., 2014; Scheumann et al., 2014), a lot of which humans share with other animal species. Although many of these non-music-specific abilities are necessary for music perception, they are not sufficient to experience music in its whole structural and meaningful form. After all, only *Homo sapiens* among all living primates is able to vocalize syntactically organized and culturally transmitted sound patterns based on rhythm and pitches. Of course, this does not mean that human musicality evolved from scratch. On the contrary, many preadaptations among our ancestral primates must have existed to allow natural selection to design music-like behavioral traits in some evolutionary lineages of primates. A good example of such a behavioral trait can be found in gibbon songs (Geissmann, 2000; Merker, 2000). This opens the possibility that even the common ancestor of great and lesser apes was endowed with the abilities to allow some form of singing (Jordania, 2014). However, taking into account that gibbon songs are under strong genetic control rather than being vocally learned calls (Brockelman and Schilling, 1984), it is reasonable to assume that the evolution of culturally flexible musical behavior among hominins was influenced by additional factors. This means that human-specific musical brain equipment had to have evolved relatively recently after the split between the common ancestor of chimpanzees and hominins. This equipment must have allowed our ancestors to use sounds as culturally heritable units. This ability let our ancestral hominins to partially free their vocalizations from affective calls (Ackermann et al., 2014). The unchained vocal calls became based on discrete units which became the units of cultural inheritance (Savage, 2019). Moreover, the sequences of these units, being culturally transmitted information, started to be the objects of modification. This is the evolutionary stage where plasticity and creativity started to act in the domain of vocal communication.

### Environmental Sounds, Speech, and Music

Although vision is the dominant sense among primates (in humans for instance the processing of visual signal involves a remarkably bigger amount of neural tissue than the processing of auditory stimuli; Deutsch, 2019), hearing is still an important source of information about the environment for humans and the same must have been true for hominins. Therefore, it is not surprising that auditory information serves as an additional clue to navigate in the world (Horowitz, 2012). However, apart from inferring information about the environment, primates also use sound to transmit signals concerning their intentions (Hauser and Konishi, 1999; Hauser, 2000). Since primates are able to auditory learn, i.e., to associate sounds with referential meaning (Wright et al., 1990) and chimpanzees even modify and use sounds as sound symbols (Watson et al., 2015), it is reasonable to assume that hominins were able to use sounds intentionally as a medium of communication. It has been proposed that the increasing role of sociality among hominins that belong to the lineage leading to *Homo sapiens*, created an evolutionary pressure for the development of intentional vocalizations, i.e., speech and singing (Dunbar, 1996). The conscious intentionality of vocal expressions imposes a set of new properties on the production and processing of sound. First of all, the coding of meaning in vocally produced sounds necessitates the vocal control over the acoustic features of vocalized sounds. Secondly, the acoustic features of sound must be linked together with meaningful mental categories. Thirdly, these mental categories must be processed separately in order to avoid confusion in the process of decoding information. Fourthly, auditory perception should be biased in favor of acoustic features that are crucial for the conspecific intentional vocalizations. Fifthly, the intentional vocalizations should be ductile enough to code new meaningful elements. Finally, these elements should be prone to be learnable, i.e., to be culturally inherited. All these properties characterize the production and processing of both speech and singing.

Human vocal learning is especially efficient as far as the speech and singing features are concerned (Jackendoff and Lerdahl, 2006). The mental categories of words in speech and musical pitches and rhythms in music are mapped into conceptual meaning (Bickerton, 2009) and preconceptual emotional (Podlipniak, 2020) and kinetic impressions (Grahn and Rowe, 2009, 2013; Levitin et al., 2018), respectively. Although neuroimaging studies show that certain brain areas are involved in the processing of both language and music (Fedorenko et al., 2009; Kunert et al., 2015) there are also neural structures that are activated when only one of these two phenomena is processed (Rogalsky et al., 2011; Perruchet and Poulin-Charronnat, 2013; Norman-Haignere et al., 2015). This dissociation indicates the existence of language and music specific networks. The perceptive preference for the specific features of speech and music is observed in children right after birth (Fassbender, 1996; McMullen and Saffran, 2004; Perani et al., 2010; Brandt et al., 2012). Finally, both language and music represent a recursive open system (Merker, 2002; Pinker and Jackendoff, 2005), which means that humans are able to produce an enormous number of new and original musical and speech phrases. Importantly, in contrast to many songbird species, humans are prone to learn and modify these phrases during their whole life. However, some elements of speech and music such as phonemes and pitch intervals seem to be easier to learn in childhood whereas some, such as new words or new musical phrases, are equally easy to learn during adulthood (Trainor, 2005; White et al., 2013; Li et al., 2014; Friedmann and Rusou, 2015; Birdsong, 2018). This suggests that learning plasticity is a complex ability that consists of selective constraints which act differently at different stages of development.

### Rhythm, Movement, Pitch, and Vocal Learning

The music specific elements of musical code are discrete rhythm measures and pitch intervals. Since both these musical features have been recognized as musical universals (Brown and Jordania, 2011; Savage et al., 2015; Trehub, 2015; Mehr et al., 2019) it is reasonable to assume that music communication is based on sounds interpreted by human minds in term of discrete pitches and rhythms. Moreover, rhythm measures and pitch intervals are crucial for musical syntax. However, discrete pitches and rhythms can exist independently as in music based solely on rhythm, as well as in non-metrical music, e.g., in some oral traditions of Gregorian chanting, respectively. This indicates that the processing of musical pitch and the processing of musical rhythm are in fact separate abilities. Therefore, human musicality cannot be treated as a monolithic entity or as a product of one evolutionary episode. Indeed, many scholars have proposed the multistage scenarios of music’s origins (Mithen, 2006). In the majority of these scenarios, musical rhythm is usually indicated as evolutionarily more ancient than musical pitch.

Musical rhythm is organized in a framework based on the sensation of periodicity known as musical pulse. Additionally, this framework is usually organized into hierarchical beat patterns called meters (London, 2012). This means that our perception of musical rhythm is based on precise mental expectations concerned with when sounds may occur (Huron, 2006). The evolutionary roots of musical rhythm have often been searched for in the coupling between auditory signals and movement. The main hint for such a claim is that humans spontaneously synchronize their movements with musical beat, the perception of which is based on the sensation of musical pulse. It is also known that the processing of isochronous musical stimuli involves the activity of motor brain areas including cortico-striatal loops (Geiser et al., 2012). This can additionally suggest that our perception of musical meter is at least partly based on motor experience (Repp, 2007). Therefore, rather than auditory, music is an auditory-motor phenomenon which along with its time dependence, makes music experience and creativity unique (Bashwiner, 2018) among other human sound expressions. The ability of auditory-motor synchronization affects not only one individual, but also the social level of behavior by allowing people to move together in synchrony in response to music. It has been also proposed that apart from movement synchronization, collective listening to music leads to the alignment of brain states (Bharucha et al., 2011).

Although the human ability to synchronize movements with beat seems to be exceptional among primates, there are studies which indicate that motor-beat synchronization restricted to 600ms periodicity can be achieved by chimpanzees (Hattori et al., 2013, 2015). Chimpanzees are also prone to rhythmic swaying in response to auditory stimuli and the beat rate of these stimuli influences the periodicity of chimps swaying in a bipedal position (Hattori and Tomonaga, 2020). These observations suggest that primitive auditory-motor coupling was present in the last common ancestor of *Homo sapiens* and chimpanzees. This coupling could have probably been one of the ancient preadapations for human musicality. However, while auditory-motor synchronization among chimpanzees is restricted to a narrow scope of tempi (Hattori et al., 2013), humans are able to spontaneously synchronize with isochronous auditory stimuli in a multi-timescale (Parncutt, 1994; Toiviainen et al., 2010; Patel and Iversen, 2014). The widening of a beat extraction timescale is a good example of a change that enables plasticity in the domain of beat perception and production. Therefore, while the first hominins were most probably unable to use sounds in different timescales, the broadening of beat sensitivity among the next generations of hominins opened the window of creativity in the domain of rhythm. Although the change of tempo does not seem to be a very creative task for humans today, it must have been a great achievement for hominins. The extension of a beat extraction timescale was also a necessary condition for the development of rhythm measures quantification. As a result of this, the perception of rhythm measures in terms of simple integer ratios is nowadays a cross-cultural universal feature of human music perception (Jacoby and McDermott, 2017). However, having many rhythm measures available, hominins developed a medium of communication susceptible to cultural change and inventions. Therefore, on the one hand the abilities to align movements with beat and to quantify rhythm measures in terms of simple integer ratios are the examples of canalized skills. At the neuronal level the ability of beat extraction is based on cortico-striatal loops, especially on connections between the supplementary motor area, the premotor cortex, auditory cortex and the putamen (Grahn and Rowe, 2009). This suggests that the evolution of hominins’ auditory-motor synchronization has been achieved by the development of these cortico-subcortical connections. On the other hand, the possibility of sensing different periodicities as musical pulse along with flexibility in the use of rhythm measures became an area of behavioral plasticity, due to the fact that both the striatum and the neocortex belong to the major sites of synaptic plasticity in the brain enabling learning (Pascual-Leone et al., 2005; Surmeier et al., 2009; Perrin and Venance, 2019). Both the ability of beat extraction and the sense of different periodicities most probably became the first capacities which enabled divergent musical thought composed of rhythmic kernels of musical thinking (Webster, 2002). Note however, that neither all canalized rhythmical skills, nor the whole scope of plasticity in the domain of musical rhythm appeared at the same time in the evolution. In order to allow hominins to evolve into a rhythmically creative species like *Homo sapiens*, a chain of successive behavioral changes had to take place. The first step in this process had to be a gradual extension of beat extraction timescale. It is possible that this step could have been achieved previously by means of behavioral plasticity. However, the scope of creativity in the domain of beat extraction must have been constrained by the restricted range of possible periodicities. This range must have gradually broadened until the appearance of anatomically modern humans in whom it reaches the span of 300–900 ms (Toiviainen et al., 2010). Importantly, beat extraction is prone to enculturation which additionally restricts creativity (London, 2012). Even today people from different cultures are biased to sense different pulse of the same music sequence depending on their cultural background (Agawu, 1987). Nonetheless, this kind of culturally induced constraint can be overcome by a creative individual thanks to often conscious convergent thinking that interplays with divergent thinking (Webster, 2002).

Another supposed area of the interplay between plasticity and canalization in the evolution of music creativity is the domain of musical pitch. Pitch in music is usually perceived as a sequence of discrete sensations representing pitch intervals (Krumhansl, 1990; Rakowski, 1999, 2009); thus, creativity in this domain may consist of composing subsequent pitches in a melody and sometimes in changing the size of pitch intervals. In contrast to the experience of musical rhythm which is based on the predictions about sound timing that are independent of the spectral characteristic of the sounds, musical pitch is sensed only if the perceived sound contains harmonics. The crucial acoustic parameter that influences the sensation of pitch is fundamental frequency (F_0_). Simply speaking, while musical rhythm tells us “when” sounds occur, musical pitch answers the question about the content of musical sequence. Not only humans use harmonic sounds as a medium of coding their intentions. Many other species, including primates, communicate using sound frequency modulation (Hauser and Konishi, 1999; Hauser, 2000; Horowitz, 2012). Continuous changes of sound frequency are parts of the affective calls of nonhuman primates (Zimmermann et al., 2013; Scheumann et al., 2014). This indicates that hominins also had to use pitch in their calls. However, a leap forward in the evolution of musical creativity was the appearance of digital (discrete) elements of vocalizations. In contrast to affective calls, the features of which are present also in human affective prosody in speech, the digital forms of human vocalizations (articulate speech and singing) are subjects to greater volitional control which gives more space for creativity.

Although not only pitch is used in a discrete form in human vocal expressions, as in the case of vowels and consonants in speech (Jackendoff, 2009), harmonic sounds must have been pivotal in the evolution of both music and speech. The appearance of digital vocal communication was possible thanks to vocal learning—the ability that allows us to vocally reproduce the acoustic parameters of the sounds that we hear (Janik and Slater, 1997; Merker, 2012). The evolution of vocal learning is also related to increased cortico-striatal connectivity (Jarvis, 2007; Fitch and Jarvis, 2013). Although humans are not equally good at vocal learning of all sounds, we are especially predisposed to imitate the distinctive elements of speech and singing. In other words, our cognitive system is biased in favor of speech formats and F_0_ of sounds in terms of their perception as well as production. Yet the volitional vocal control of F_0_ was the most important evolutionary change (Bannan, 2012) that initiated the creative use of discrete pitch. Only having the ability to sustain F_0_ of sung sounds and to master the size of vocalized pitch intervals allowed hominins to be inventive in producing original sequences of discrete pitches. Therefore, while the vocal learning biases became the canalized roots of music and speech development, the establishment of the basic discrete units of speech and music—e.g., phonemes of a particular language and discrete pitches of culture-specific musical system—represents the scope of plasticity. However, it is possible that at the ancient stages of hominins’ vocalizations, the choice of spectral cues (F_0_ or speech formants) as the distinctive features of discrete vocalization units was also established by means of behavioral plasticity. It is well known that practicing music can lead to the enlargement of and the induction of neuroplasticity in cortical areas involved in the processing of pitch information such as the planum temporale (Meyer et al., 2012; Bashwiner et al., 2016) as well as to the increased connectivity between areas involved in music processing such as the auditory, the sensorimotor, and the prefrontal cortices (Klein et al., 2016). Such neuronal mechanisms enable behavioral plasticity and could have been present to some extent in early musical hominins allowing them to create discrete vocalizations. The fact that there is a correspondence between the number of vowels in language and the number of pitches in musical scales, as well as a relationship between vowel formants and F_0_ in song based on meaningless syllables (Fenk-Oczlon, 2017) can support such a scenario. Only after a persistent use of F_0_ as a culturally learned distinctive clue for the recognition of song units did the perceptive bias in favor of musical pitch appear. From a cognitive point of view, the perceptive biases in favor of musical pitch and regularity in musical rhythm consist of an active search for relevant information ignoring irrelevant acoustic features. These biases restrict musical creativity in a similar way to phonemic constraints in language (Merker, 2006). The appearance of musical pitch extended the scope of mental categories that constitute the units of musical thoughts present in Webster’s divergent thinking phase (Webster, 2002).

## Musical Syntax as a Canalized Framework for Musical Creativity

The perceptive biases that constrained musical creativity in the domain of rhythm and pitch structure are not restricted solely to the active search for the distinctive features of musical discrete units. Music similar to language is a complex communicative tool, the structure of which is governed by syntactic rules (Lerdahl and Jackendoff, 1983). These rules mean that musical structure is perceived in terms of two types of hierarchy—rhythm hierarchy based on meter (London, 2012) and pitch hierarchy based on pitch centricity (Krumhansl and Cuddy, 2010). From this point of view music represents a generative and recursive system known as the Humboldt system (Merker, 2002), which allows people to create an enormous number of socially appreciated music sequences. This enormity of such sequences does not mean, however, that every sequence of sound can be recognized as correct. Quite the opposite, there is at least an equally big set of sound sequences that are unacceptable by a social group as faultless musical pieces. This means that even a musically untrained listener is able to recognize syntactical faults in musical sequences such as an out-of-key note in a tonal sequence (Tillmann et al., 2000; Gorzelańczyk et al., 2017). Such a tacit recognition of faults resembles the feelings that accompany native speakers when they listen to grammatical errors in their mother tongues. The main difference between music and language in this respect is that word hierarchies (grammatical relations) are conceptual. Although musical syntax, in contrast to language, is not related to propositional semantics (Lerdahl, 2013) the structural hierarchies in music are easily recognizable by means of emotional clues. It is assumed that the emotional response to sound sequences occurs as a result of fulfilling or not the predictions based on the implicit statistical learning of sound distribution in the musical environment.

The fact that musical syntax is learned implicitly and spontaneously in childhood suggests the existence of developmental predispositions related to this task. It has been hypothesized that the ability to recognize pitch center (Podlipniak, 2016), as well as to organize musical pitch in a syntactic way (Podlipniak, 2020), evolved by the means of the Baldwin effect, which means that after cultural invention of pitch hierarchy it was overtaken by genetic control. Regardless of whether it is true or not, the fact that tonal organization of music is prevalent across musical cultures (Mehr et al., 2019) strengthens the view that human proclivity to implicitly learn the rules of pitch distribution in music is a result of the process of canalization. After all, the prevalence of tonality among musical cultures across the world, which are enormously diversified in respect of other musical traits, indicates that pitch syntax must have become a stable musical feature. It has been either culturally transmitted from an ancient common ancestral culture or it develops independently thanks to some genetic proclivities. The same reason can explain the universality of hierarchical organization of musical rhythm. As a result, musical syntax usually consists of pitch and rhythm hierarchies. Therefore, both explanations are consistent with the claim that the use of a pitch-rhythm framework for musical syntax is an example of a canalized behavioral trait of *Homo sapiens*. To some extent musical syntax is like the grammar of a mother tongue. As the tacit knowledge of grammar restricts the possible word flexion and order in sentences, the musical syntax puts constraints on musical expressions. In other words, musical syntax answers the question when and what kind of pitch is acceptable. However, there is not only one answer to this question. Moreover, the permanently changing social environment means that the aesthetic preferences are also changing. As a result, different tunes may bring the house down in different times. Having many possible melodic variants that are congruent with the syntactic rules of a particular music style, and that can simultaneously satisfy the aesthetic preferences of a given social group opens space for creativity. This kind of creativity is often called “combinational” (Boden, 2004, p. 3) since the innovation represents a new combination of sounds within a framework of the present syntactic rules. Sometimes, however, an individual is able to create sound sequences that surpass the existing rules and to implement new ones. This kind of creativity is called “exploratory” (Boden, 2004, p. 4) as it explores formerly unknown rules of organization. It is possible that the neurological structures that are not music-specific, such as the default mode network, play some role in such innovative thinking. In both cases, however, the creative individuals must be familiar with the aforesaid implicit knowledge which means that they are constrained by the existing implicitly learned rules. After all, changing the rules necessitates the knowledge of what has changed. The new rules, if socially accepted, become implicitly learned by the next generations of listeners until new innovative rules are created and socially accepted. This endless process is a good example of cultural evolution which runs faster than gene-culture coevolution. However, in the long run, even in a fast-changing culture, some features can become canalized. Canalization opens the coevolutionary concatenation of events. The example of a ubiquitous syntactic pitch-rhythm framework of music suggests that human musicality has become a subject of canalization. However, although musical creativity, being a force that leads to musical change, acts against canalization it is at the same time a product of it. Without the canalized perceptual traits such as rhythm measures, pitch intervals, etc. musical creativity would be devoid of a well-defined space.

## Conclusion

According to the presented view, musical creativity did not only appear in the course of hominin evolution but also became a driving force of the gene-culture coevolution of human musicality. Being an inseparable part of a cultural plastic change, creativity in the domain of pitch and rhythm gave diversity which became the subjects of cultural selection. Only such circumstances opened space for the canalization of pitch intervals and rhythm measures as discrete musical units that dominated human musicality for millennia. Moreover, since the default experience of musical pitch and rhythm is preconceptual, their appearance most probably preceded the evolution of the conceptual mind. This suggests that creativity in the domain of musical pitch and rhythm represents a domain specific ability in contrast to creativity that operates in the conceptual mind. Of course, it does not exclude the possibility that people can be creative in music using conceptual resources. This is especially possible as part of Webster’s aforementioned creative thinking process at the level of convergent thoughts (Webster, 2002). After all, professional musicians are able to learn pitch intervals and rhythm schemes as precise defined concepts and categorically perceived entities. This task necessitates, however, strenuous and time-consuming learning, suggesting the crucial role of phenotypic plasticity in this process. Moreover, musical phrases can be the source of associations that can be used in creative composing which can be consciously incorporated into analytical thinking leading to aesthetic decisions. Nevertheless, music-specific creativity operates mainly in the realm of tonal music, whereas general creativity can be especially desirable in “sound arts” and other kinds of music that abandon rhythm and pitch syntaxes.

The proposed view that musical creativity is linked to gene-culture coevolution emphasizes the important role of individual invention in the process of generating cultural variability. After all, an increasing number of song variants achieved by individuals’ creativity extends the scope of a cultural environment to be selected. Independent of this variability the social success of creative individuals could facilitate natural selection of those individuals whose creativity meets the social requirements. As a result, over a long period of time human musicality appears to be a permanently changing capacity influenced by both social aesthetic trends and our predispositions to be creative in the domain of music. This implies that musical creativity is a part of human nature which means that musical creative thoughts should be achievable by the majority of people. Although the proposed evolutionary scenario is speculative there are some possible areas of study which can explore its implications. The search for genetic predispositions of musicality is the most promising scope of research in this respect. Another area research that can help to answer the question about the role of plasticity and canalization in evolution musical creativity is the neuroscience of music. The recognition of the limits of neural plasticity related to the processing of musical pitch and rhythm could shed some light on the specificity of creativity in the domain of music.

## Data Availability Statement

The original contributions presented in the study are included in the article further inquiries can be directed to the corresponding author.

## Author Contributions

The author confirms being the sole contributor of this work and has approved it for publication.

## Conflict of Interest

The author declares that the research was conducted in the absence of any commercial or financial relationships that could be construed as a potential conflict of interest.
